# Superfluid-Insulator Transition unambiguously detected by entanglement in one-dimensional disordered superfluids

**DOI:** 10.1038/s41598-019-51986-0

**Published:** 2019-10-25

**Authors:** G. A. Canella, V. V. França

**Affiliations:** Institute of Chemistry, São Paulo State University, 14800-090 Araraquara, São Paulo Brazil

**Keywords:** Phase transitions and critical phenomena, Quantum fluids and solids, Superconducting properties and materials, Quantum information

## Abstract

We use entanglement to track the superfluid-insulator transition (SIT) in disordered fermionic superfluids described by the one-dimensional Hubbard model. Entanglement is found to have remarkable signatures of the SIT driven by *i)* the disorder strength *V*, *ii)* the concentration of impurities *C* and *iii)* the particle density *n*. Our results reveal the absence of a critical potential intensity on the SIT driven by *V*, i.e. any small *V* suffices to decrease considerably the degree of entanglement: it drops ∼50% for *V* = −0.25*t*. We also find that entanglement is non-monotonic with the concentration *C*, approaching to zero for a certain critical value *C*_*C*_. This critical concentration is found to be related to a special type of localization, here named as *fully-localized state*, which can be also reached for a particular density *n*_*C*_. Our results show that the SIT driven by *n* or *C* has distinct nature whether it leads to the full localization or to the ordinary one: it is a first-order quantum phase transition only when leading to full localization. In contrast, the SIT driven by *V* is never a first-order quantum phase transition independently on the type of localization reached.

## Introduction

Localization and superconductivity are phenomena with opposite features. In a superconductor the effective attractive interactions lead to long-range electronic order, allowing supercurrents without resistance. In contrast, localized systems have no flux of electrical charge, thus behaving as insulators. Hence nanostructures, ultracold atoms and condensed-matter systems exhibiting both, superconductivity and localization, represent a rich scenario with interesting and unconventional properties.

According to Anderson’s theorem^1^, localization is expected to occur in strong or moderate disordered systems. In the regime of strong disorder, the electronic wavefunctions become localized, transforming metals or superconductors in insulators^2^. The latter case – the so-called superfluid to insulator transition (SIT)^3–5^ – should be driven either by increasing the disorder strength or by lowering the particle density. The SIT is marked by the decrease of the superfluidity fraction^6^ and a distinct non-monotonic behavior of the condensate fraction with disorder intensity^7^.

Disorder remains a current topic, with possible implications to quantum information technologies^8–12^, while SIT driven by disorder is very important for being considered a paradigmatic quantum phase transition in many-body systems^13–15^. Understanding the SIT can reflect in better comprehension of many complex superfluid systems such as high-*T*_*c*_ superconductors (intrinsically disordered^5^), superconducting nanowires, amorphous superconductors, ultracold atomic gases, and molecules^16–19^.

Most of the experiments have focused on weak or non-interacting bosonic systems^20–24^. Theoretically, one faces the challenge of treating many-body interactions and the many realizations typically needed for describing disorder. Although the properties of the SIT should not depend on the entity used to track the transition^25^, some quantities may not be sufficiently sensitive to the SIT, and thus do not present any distinct behavior.

In this sense entanglement measures appear as good candidates to pursue the SIT, since they have been successfully used to detect quantum phase transitions in several contexts^26–29^. Localization has been investigated via entanglement in metals^30–33^, bosonic systems^34–38^, Bose-Fermi mixtures^39^ and spinless fermions^40–42^.

However, none of the previous studies *i)* has considered fermionic superfluid systems (with both charge and spin degrees of freedom) and *ii)* none has found entanglement as an unambiguous signature of the SIT: the typical fingerprints of a quantum phase transition (non-monotonicity, discontinuity or saturation) were missing. Furthermore, important features of the SIT are in current debate, as the existence or not of a critical disorder intensity for localization in 1D and 2D systems^5,43–47^, as well as the nature of the transition, whether it is a more pronounced transition or a crossover^6,48^.

Here we report entanglement as an unequivocal signature of the SIT in one-dimensional disordered fermionic superfluids. We explore a unique opportunity to better understand the SIT through standard density-functional theory (DFT)^49^ applied to the Hubbard model, within a specially designed density functional for the linear entropy^50^. This approach allows us to generate many realizations for each set of parameters, comprising a huge amount of data, what would be prohibitive via exact methods as density-matrix renormalization group.

Our main results can be summarized as follows: (a) Entanglement detects the SIT driven by all the parameters: disorder strength, concentration of impurities and particle density. (b) The SIT driven by the disorder strength requires no critical potential intensity to occur: any small disorder suffices to drive the transition. (c) For sufficiently strong disorder strength, a particular type of localization emerges at a critical *C*_*C*_ or at a critical *n*_*C*_. This state is marked by null entanglement and therefore is here named as *fully-localized state*. (d) The SIT driven by the concentration and by the particle density are found to be quantum phase transitions of first order only when the system reaches the fully-localized state. In contrast, the SIT driven by the disorder strength is not a first-order quantum phase transition for both, ordinary and full localization.

## The Theoretical Model and Computational Methods

We consider one-dimensional superfluids at zero temperature, where classical fluctuations are absent, described by the fermionic Hubbard model:

1 $$H=-\,t\sum _{ < ij > \sigma }{\hat{c}}_{i\sigma }^{\dagger }{\hat{c}}_{j\sigma }+U\sum _{i}{\hat{n}}_{i\uparrow }{\hat{n}}_{i\downarrow }+\sum _{i\sigma }{V}_{i}\,{\hat{n}}_{i\sigma }\mathrm{.}$$

Here *U* < 0 is the attractive on-site interaction, *t* is the hopping parameter between neighbor sites $< ij >$ and ${V}_{i}$ is the external potential, used to simulate the disorder. The density operator is ${\hat{n}}_{i,\sigma }={\hat{c}}_{i,\sigma }^{\dagger }{\hat{c}}_{i,\sigma }$, where ${\hat{c}}_{i,\sigma }^{\dagger }$ (${\hat{c}}_{i,\sigma }$) is the creation (annihilation) operator of fermionic particles with *z*-spin component $\sigma =\uparrow \,,\downarrow$ at site *i*, $n=N/L$ is the average density or filling factor, $N={N}_{\uparrow }+{N}_{\downarrow }$ the total number of particles and *L* the chain size. We consider spin-balanced populations, ${N}_{\uparrow }={N}_{\downarrow }$, $L=100$, open boundary conditions, $U=-\,5t$ and $n=0.8$, unless otherwise stated.

Our disordered superfluids are characterized by a certain concentration $C=({L}_{V}/L)\times \mathrm{100 \% }$ of pointlike impurities^47,50^, each one of strength *V* randomly placed along the chain, such that there are ${L}_{V}$ sites with impurity *V* and $L-{L}_{V}$ non-impurity sites ($V=0$). For each set of parameters, $(C,V;U,n)$, we generate $M=100$ samples to avoid features due to specific impurity configurations. Then any property for that given set of parameters is averaged over *M* (see Supplementary Material online, Fig. S4). This implies however in a huge amount of data, which would be prohibitive with numerically exact methods, such as density-matrix renormalization group calculations.

We apply instead standard DFT^51^ techniques to solve the model — the self-consistent Kohn-Sham scheme within a local density approximation for the exchange-correlation energy (for a review on the regime of accuracy of this formalism see for example^50,52–55^) — obtaining the ground-state energy and the density profile, with fair precision (∼1% for chains of this size). To quantify the degree of entanglement in such superfluids we consider the single-site entanglement quantified by the linear entropy

2 $${ {\mathcal L} }_{i}=1-Tr({\rho }_{i}^{2}),$$

which corresponds to the linear term of the von Neumann entropy — a well-defined entanglement measure for bipartite pure systems^56^. Here ${\rho }_{i}=T{r}_{L-1}(\rho )$ is the reduced density matrix of site *i* obtained by tracing the total density matrix with respect to the remaining $L-1$ sites. We adopt a specially designed density functional for Eq. (2) ^50^, which is then used as input in a local density approximation, following the protocol proposed in ref. ^57^, to obtain the average single-site entanglement of the inhomogeneous disordered chains.

## Results

The SIT is expected to be driven either by enhancing the disorder strength (at a constant density) or by lowering the particle density (at a constant disorder)^5^. Nevertheless, in our pointlike type of disorder^47,50^ (see refs. ^36,37,39–42,58,59^. for other potential landscapes) the concentration of impurities is an additional important parameter whose impact on the transition remains to be investigated. Thus we analyze the SIT tuned by disorder strength, impurities’ concentration and particle density.

### SIT driven by disorder strength

**Figure 1:**
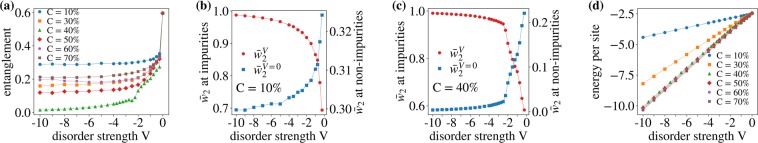
SIT driven by the disorder strength *V*: entanglement ${\mathcal L}$ (**a**), average double-occupation probabilities at impurity (${\bar{w}}_{2}^{V}$) and non-impurity (${\bar{w}}_{2}^{V\mathrm{=0}}$) sites for *C* = 10% (**b**) and *C* = 40% (**c**), and per-site ground-state energy (**d**).

We start by tracking the transition across the impurities’ strength *V* at a fixed density for several concentrations. Figure 1a reveals that entanglement quickly saturates with *V*: ${\mathcal L}$ drops already ∼50% for $V=-\,0.25t$ for any *C*. This entanglement saturation is an unequivocal signature of the SIT: the initial superfluid, at *V* = 0, is transformed into strongly-coupled localized dimers, which now make the system an insulator with no further effect by enhancing |*V*|. Also, the quick decreasing of ${\mathcal L}$ for small *V* supports the absence of a critical disorder intensity for the SIT driven by disorder strength: even a weak potential drives the system from the superfluid state to the localized one.

Figure 1a also shows that entanglement is non-monotonic with the concentration: for a fixed *V*, ${\mathcal L}$ decreases with *C*, approaching to zero at a certain critical value *C* = *C*_*C*_ (*C*_*C*_ = 40% in this case), and thus increasing again for $C > {C}_{C}$. Notice that, although any concentration leads the system to localization, marked by the saturation of ${\mathcal L}$ for $V\to -\,\infty$, two features suggest that at *C* = *C*_*C*_ there is a special type of localization. First, the fact that for weak disorder ($-3t < V\mathrm{ < 0}$) entanglement behaves with $V$ differently at *C* = *C*_*C*_ when compared to all the other *C*′*s*. Secondly, *C* = *C*_*C*_ is the only case where ${\mathcal L} \to 0$ for $V\to -\,\infty$. By analysing several systems^60^ in order to understand what is special at *C* = *C*_*C*_, we find that *C*_*C*_ is the concentration for which the number of impurity sites, ${L}_{V}$, corresponds exactly to the total number of pairs, $N\mathrm{/2}$, thus leading to the relation ${C}_{C}=(n\mathrm{/2)}\times \mathrm{100 \% }$. This suggests that at *C* = *C*_*C*_ the strongly coupled dimers are *fully localized* at the impurities sites which, for *V* < 0, are the most attractive ones.

This is confirmed by the average double-occupation probabilities at impurity sites, ${\bar{w}}_{2}^{V}={\sum }_{i}\langle {\hat{n}}_{i\uparrow }{\hat{n}}_{i\downarrow }\rangle /{L}_{V}$, and non-impurity sites, ${\bar{w}}_{2}^{V\mathrm{=0}}={\sum }_{i}\langle {\hat{n}}_{i\uparrow }{\hat{n}}_{i\downarrow }\rangle /(L-{L}_{V})$. For *C* ≤ *C*_*C*_ (Fig. 1b), the dimers are in larger number than impurity sites, thus there are localized dimers at impurity sites (${\bar{w}}_{2}^{V}\to 1$ for $V\to -\,\infty$) but also some delocalized pairs at non-impurity sites (${\bar{w}}_{2}^{V\mathrm{=0}}$ saturates in a finite value, ∼0.3). For *C* ≥ *C*_*C*_ (Fig. 1c), the system remains with a certain degree of delocalization since there are more impurity sites than dimers, but the non-impurity sites are empty (${\bar{w}}_{2}^{V\mathrm{=0}}\to 0$ for $V\to -\,\infty$).

Thus the full localization at *C*_*C*_ represents a well defined state with unitary probability of pairs at impurity sites and null probability of pairs at non-impurity sites, which is then characterized by ${\mathcal L} \to 0$ (for $V\to -\,\infty$), in contrast to the ordinary localization in which ${\mathcal L}$ saturates, but at finite values. Interestingly though the ground-state energy, shown in Fig. 1d, has no special behavior (see Supplementary Material online, Fig. S1), thus suggesting that the SIT driven by *V* is smooth, a second-order quantum phase transition or simply a crossover^61^, independently on which type of localization is reached.

### SIT driven by concentration of impurities

**Figure 2:**
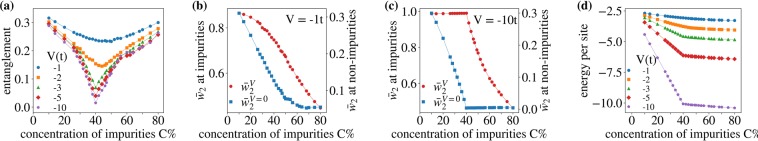
SIT driven by the impurities’ concentration *C*: entanglement ${\mathcal L}$ (**a**), average double-occupation probabilities at impurity (${\bar{w}}_{2}^{V}$) and non-impurity (${\bar{w}}_{2}^{V\mathrm{=0}}$) sites for $V=-\,t$ (**b**) and $V=-\,10t$ (**c**), and per-site ground-state energy (**d**).

Next we monitor entanglement across the disorder concentration, as shows Fig. 2a for weak, moderate and strong disorder intensities. We find that a minimum disorder strength ${V}_{min}$ is needed for the existence of the full localization at the critical *C*_*C*_, marked by a sudden non-monotonicity of the entanglement and by ${\mathcal L} \to 0$ for $V\to -\,\infty$. For $|V| < {V}_{min}$ (in this case ${V}_{min}\sim 3t$), we observe a very distinct behavior of entanglement as a function of *C*: the non-monotonicity is not abrupt, the minimum of entanglement is larger than zero and it does not occur at *C* = *C*_*C*_ = 40%.

We interpret this distinct behavior for $|V| < {V}_{min}$ as a *frustrated full localization*: *V* is not sufficiently strong to fully localize the dimers at the impurity sites. This is indeed confirmed by the average double occupancy at impurity and non-impurity sites, shown in Fig. 2b: ${\bar{w}}_{2}^{V\mathrm{=0}}$ is always above zero for $V=-\,t$. In contrast, Fig. 2c shows that for $V=-\,10t$ the fully-localized state is reached at *C*_*C*_: ${\bar{w}}_{2}^{V}\approx 1$ for *C* ≤ *C*_*C*_, and ${\bar{w}}_{2}^{V\mathrm{=0}}\to 0$ for *C* ≥ *C*_*C*_.

For $|V| > {V}_{min}$, the ground-state energy, shown in Fig. 2d, reveals the nature of the transition from the ordinary to the fully-localized state driven by *C*: it has a discontinuous first derivative at *C* = *C*_*C*_ (see Supplementary Material online, Fig. S2), thus characterizing a first-order quantum phase transition^61^. For $|V| < {V}_{min}$ the energy is smooth, with no discontinuity neither on the first nor on the second derivative, suggesting no transition at all, what is consistent with the absence of the fully-localized state. The existence of this *V*_*min*_ for full localization explains the distinct behavior of *C* = *C*_*C*_ for small *V*, observed in Fig. 1a.

### SIT driven by particle density

**Figure 3:**
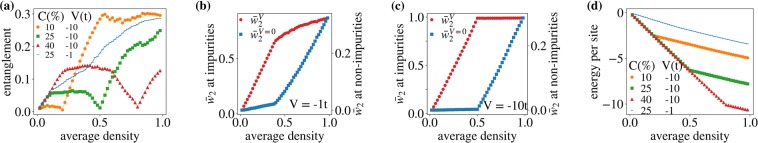
SIT driven by the particle density *n*: entanglement ${\mathcal L}$ (**a**), average double-occupation probabilities at impurity (${\bar{w}}_{2}^{V}$) and non-impurity (${\bar{w}}_{2}^{V\mathrm{=0}}$) sites for $V=-\,t$ (**b**) and $V=-\,10t$ (**c**), and per-site ground-state energy (**d**).

Finally, we track the transition across the average particle density for weak and strong disorder intensities, as shows Fig. 3a. For sufficiently strong *V*, all the features observed before at ${C}_{C}\,=\,\mathrm{100(}n\mathrm{/2) \% }$ are now found at the critical density *n*_*C*_ = 2*C*/100: entanglement has a sudden non-monotonicity at *n* = *n*_*C*_, the system is fully localized at ${n}_{C}$ with ${\mathcal L} \to 0$ (for $V\to -\,\infty$), and ordinarily localized with constant ${\mathcal L} \, > \,0$ for *n* < *n*_*C*_. We find however that, from the fully-localized state (at *n* = *n*_*C*_) to the ordinary localization (for *n* < *n*_*C*_), entanglement does not increase much and has a plateau. This reflects the fact that, for small densities, disorder hampers the connections among the localized dimers^5,62^ due to the larger average distance among them. Consistently, the plateau is broader and with higher ${\mathcal L}$ for larger concentrations, since the average distance among dimers decreases.

For weak disorder, *V* = −*t*, none of the features is observed, entanglement simply decreases monotonically with the density. This confirms the existence of a minimum disorder intensity *V*_*min*_ for the full localization also driven by *n*. Both, the double occupancy and the energy, shown in Fig. 3b–d, corroborate the fact that the system is successfully driven by *n* to the fully-localized state for *V* = −10*t*, while there is a frustration of the full localization for weak disorder ($V=-\,t$). The analysis of the first energy derivative (see Supplementary Material online, Fig. S3) reveals that while the SIT driven by *n* is also a first-order quantum phase transition when leading to the fully-localized state, it is not when leading to the ordinary localization.

The double occupancy is also useful to distinguish the nature of the SIT itself: the probabilities change smoothly *i)* when the SIT is driven by *V* (Fig. 1b,c) and *ii)* for weak disorder, |*V*| < *V*_*min*_, when the SIT is driven by *n* or *C* (Figs 2b and 3b). In contrast the probabilities change abruptly for strong disorder strength with the SIT driven either by *C* (Fig. 2c) or *n* (Fig. 3c).

## Conclusion

In summary, we find that entanglement is a reliable witness of the SIT driven by all the parameters: disorder strength *V*, impurities’ concentration *C* and particle density *n*. In all cases we find two types of localization: the ordinary localization, characterized by finite entanglement, and the full localization, with null entanglement (for *V* → −∞). When the system is led to the ordinary localization: there is no minimum disorder strength required to the SIT and it is not a first-order quantum phase transition, independently on the driving parameter *V*, *C* or *n*. In contrast, the full localization only occurs at a critical concentration (${C}_{C}\mathrm{=100(}n\mathrm{/2) \% }$) or equivalently at a critical density (*n*_*C*_ = 2*C*/100), and the features depend on the driving parameter: when driven by *V*, any small disorder suffices to drive the SIT and it is not a first-order transition; when driven by either *C* or *n*, there is a minimum *V*_*min*_ required to the full localization and it is a first-order quantum phase transition.

Also current controversial debates – concerning *i)* entanglement as a reliable witness of the SIT, *ii)* the nature of the transition and *iii)* the existence or not of a critical disorder strength – may be elucidated in the light of our results. Previous works investigating entanglement did not find an unambiguous signature of the SIT, however they have focused on the SIT driven by the disorder strength *V*, which has been proved here not to be a first-order quantum phase transition, even when leading the system to the fully-localized state. We have also showed that the transition classification and the existence of *V*_*min*_ depend on whether the system is led to ordinary or full localization, and to the best of our knowledge such distinction has not been considered before. Additionally, as the experimental detection and characterization of entanglement via several protocols have been a current achievement^34,63–67^, our results could contribute to the detection of quantum phase transitions in experiments.

The impact of density, interaction and harmonic confinement – essential for simulating cold atoms experiments – on the SIT has been investigated elsewhere^60^. Non-local functionals for the linear entropy may be implemented to investigate the SIT via DFT calculations in more general potential landscapes.

## Supplementary information

Supplementary Material
