# Supplementary material for: Superfluid-Insulator Transition unambiguously detected by entanglement in one-dimensional disordered superfluids
Source: Sci Rep. 2019 Oct 25;9:15313. doi: 10.1038/s41598-019-51986-0 (PMC6814829; doi:10.1038/s41598-019-51986-0)
Supplement: Supplementary file 1 — Supplementary Material [file 41598_2019_51986_MOESM1_ESM.pdf]

Supplemental Material: Superfluid-Insulator Transition unambiguously detected by entanglement in one-dimensional disordered superfluids

Guilherme A. Canella[1] and Vivian V. França[1]
[1] *Institute of Chemistry, São Paulo State University, 14800-090, Araraquara, São Paulo, Brazil*

The energy derivatives are obtained via $f'_i \approx \frac{f(x_i)-f(x_{i-1})}{h}$ and $f'_i \approx \frac{f(x_{i+1})-f(x_i)}{h}$ at the boundaries, and by $f'_i \approx \frac{f(x_{i+1})-f(x_{i-1})}{2h}$ elsewhere, with h a homogeneous stepsize between consecutive energy data.

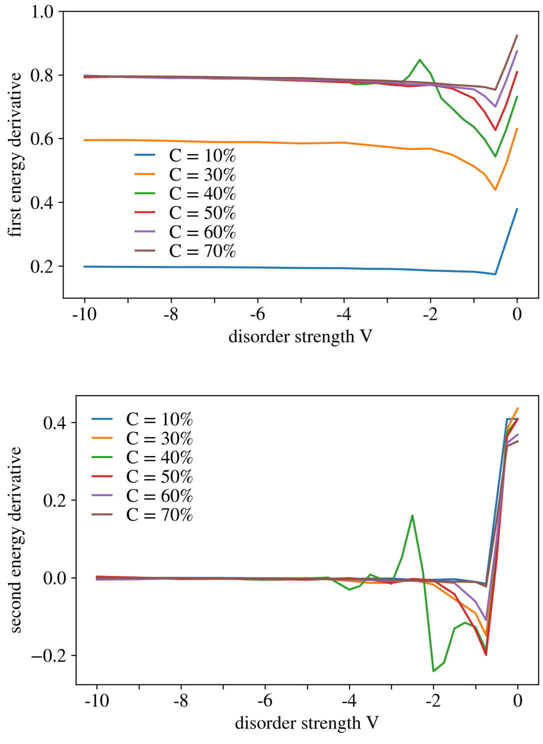

Supplementary Figure S1: First and second energy derivatives for the SIT driven by disorder strength, related to Fig.1-d in the main tex.

In Figures S1-S3 we present first and second energy derivatives for the SIT driven by the disorder intensity V (Fig.S1), by the concentration C (Fig.S2) and by the particle density n (Fig.S3). We see that across V there is no discontinuity at the first derivative for any C, while in the second derivative one could argument that there is a discontinuity for $C = 40\%$, but none for other $C's$. This supports the fact that the superfluid-insulator transition (SIT) driven by V is not a first-order quantum phase transition: it is possibly a second order when the system reaches full localization (at $C = C_C$) and a simple crossover when the system is ordinarily localized (for $C \neq C_C$). We find that across C there is a clear discontinuity at the first derivative for sufficiently strong V (for $|V| > V_{min}$, with $V_{min} \sim 3t$). This confirms that the SIT driven by C is a first-order quantum phase transition when the system reaches full localization. One can see that across n there is a unquestionable discontinuity at the first derivative for sufficiently strong V. This confirms that the SIT driven by n is a first-order quantum phase transition.

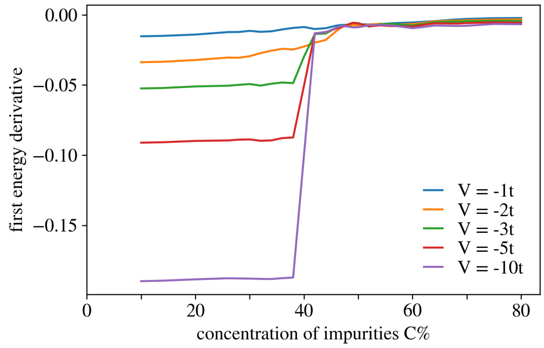

Supplementary Figure S2: First energy derivative for the SIT driven by concentration of impurities, related to Fig.2-d.

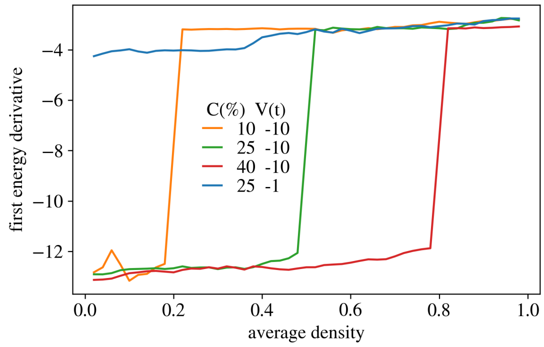

Supplementary Figure S3: First energy derivative for the SIT driven by particle density, related to Fig.3-d.

Figure S4 illustrates the convergence of the average entanglement as a function of the number of disordered samples M for several $C's$ (bellow, at and above C_C), for weak and strong disorder. One can see that for $M = 100$ – our choice in all the cases presented in the main tex – the average entanglement has converged, thus has averaged out specific impurities' configurations.

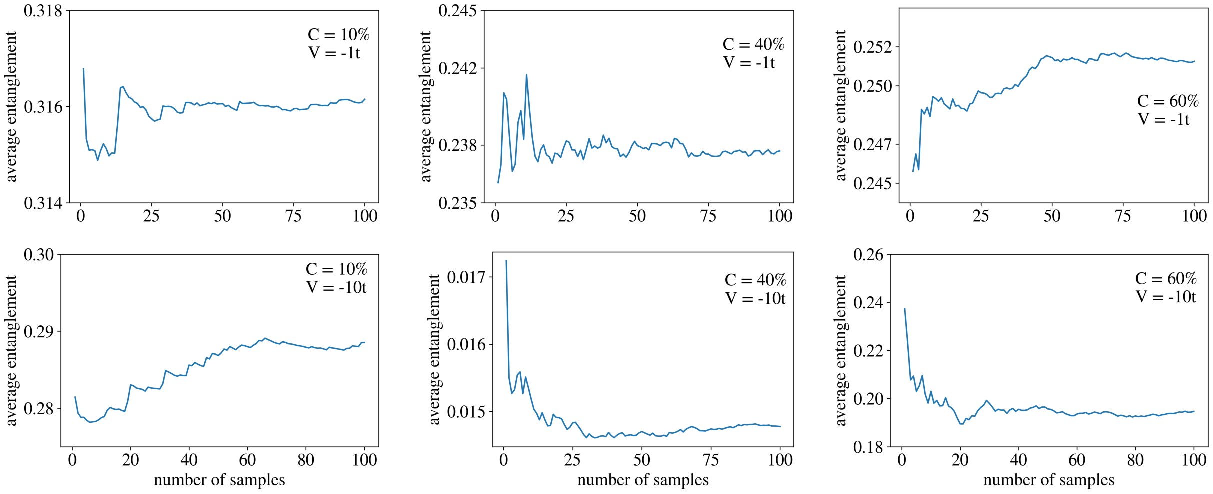

Supplementary Figure S4: Convergence of the average entanglement as the number of realizations increases: Entanglement averaged over M, the number of disordered samples, as a function of M, for $V = -t$ (upper panels) and $V = -10t$ (bottom panels), for $C < C_C$ (left), $C = C_C$ (center) and $C > C_C$ (right). In all cases $U = -5t$ and $n = 0.8$.
